# Advanced microstructure, morphology and CO gas sensor properties of Cu/Ni bilayers at nanoscale

**DOI:** 10.1038/s41598-022-16347-4

**Published:** 2022-07-14

**Authors:** Atefeh Ghaderi, Azizollah Shafiekhani, Shahram Solaymani, Ştefan Ţălu, Henrique Duarte da Fonseca Filho, Nilson S. Ferreira, Robert Saraiva Matos, Hadi Zahrabi, Laya Dejam

**Affiliations:** 1 Department of Physics, Faculty of Physics and Chemistry, Alzahra University, Tehran, 1993891167 Iran; 2 Department of Physics, Kermanshah Branch, Islamic Azad University, Kermanshah, Iran; 3 The Directorate of Research, Development and Innovation Management (DMCDI), Technical University of Cluj-Napoca, Constantin Daicoviciu St., No. 15, 400020 Cluj-Napoca, Cluj County Romania; 4 Laboratory of Synthesis of Nanomaterials and Nanoscopy (LSNN), Department of Physics, Federal University of Amazonas (UFAM), Manaus, AM 69067-005 Brazil; 5 Department of Physics, Federal University of Sergipe, São Cristovão, Sergipe Brazil; 6 Amazonian Materials Group, Federal University of Amapá (UNIFAP), Macapá, AP Brazil; 7 Postgraduate Program in Materials Science and Engineering, Federal University of Sergipe (UFS), São Cristóvão, SE Brazil; 8 Department of Physics, West Tehran Branch, Islamic Azad University, Tehran, Iran

**Keywords:** Materials science, Nanoscience and technology

## Abstract

In this study, we investigated the morphology of synthesized Cu/Ni nanoparticles in trace of carbon sources by the co-deposition process of RF sputtering and RF-PECVD methods and localized surface plasmon resonance of CO gas sensing of Cu/Ni nanoparticles. The surface morphology was studied by analyzing 3D micrographs of atomic force microscopy using image processing techniques and fractal/multifractal analyses. The MountainsMap® Premium software with the two-way ANOVA (Variance analysis) and least-significant differences tests were used for statistical analysis. The surface nano-patterns have a local and global particular distribution. Experimental and simulated Rutherford backscattering spectra confirm the quality of nanoparticles. Then, prepared samples were exposed to CO gas flue to study their gas sensor application using the localized surface plasmon resonance method. Increasing the Ni layer over Cu one shows an interesting result in both morphology and gas sensing sides. Advanced stereometric analyses for the surface topography of thin films in conjunction with Rutherford backscattering spectrometry and Spectroscopic analysis make a unique study in the field.

## Introduction

The speedy air pollution in the last decades especially because of fast industrialization encourages researchers to find out more about the importance of gas sensing. Metal nanoparticles (NPs) have shown themselves as promising materials applicable in gas sensors^1–4^ even in comparison with metal thin films to generate localized surface plasmon resonance (LSPR) which is a resonance with intense and highly confined electromagnetic fields^5–8^. Cu, as a cheap and low toxic transition metal with large applications, has been considered as an essential element by scientists and industries especially sensor manufactures^9^. On the other hand, catalysts based on Ni as a transition metal have advanced performances rather than other ones^10^. The wide range of known applications on Cu/Ni in nanoscale make them more important especially because of their structure property which does not change after alloying^11,12^.

Whereas metal NPs and their border with dielectric ambient show a significant change in LSPR so they have been applied as base materials for gas sensing^13^. When an absorption spectrum changes it means that three factors of resonance wavelength and/or absorption peak strength and/or full width half maximum can change^1–4^. In the surface of a nanostructure LSPR which is directly related to particle size, in NPs rather than thin films is an effective factor to identify molecular absorption^14^ also Ruiz et al. indicates on relation between small particles and sensing efficiency^15^.

In the case of optical gas sensing of CO, some composites like AuCo_3_O_4_^16^, Au-CuO^17^, and Au-YSZ^18^ have been reported in the literature. We can see Au as a noble metal gathered with a metal oxide to sense gas molecules that were chemically absorbed on the surface of composites but the main concern about sensors is their response at room temperature which is not provided by them.

In the last decades, atomic force microscopy (AFM) has been applied as an advanced to characterize the 3-D surface micromorphology with high nanoscale resolution^19–22^. Furthermore, stereometric, fractal/multifractal analyses^23–26^, power spectral density (PSD) functions^27^, and Minkowski functionals^28^ are modern tools to characterize the surface topography of thin films.

In the present study, Cu/Ni NPs at the trace of Acetylene (C_2_H_2_) were deposited to apply as CO gas sensors at room temperature based on LSPR absorption. Rutherford backscattering spectrometry (RBS) was used for compositional analysis and morphology-based on AFM images and MountainsMap® Premium software to process the 3D topographical maps applied to study the surface isotropy of the surface microtexture and all advanced micromorphologhy parameters. On the other hand, new scientific results that can be applied in industrial processes and are of great interest in para (CO) gas chemical sensing applications were shown. Such a combination of NPs synthesize, characterize, and application has been reported for the first time in the literature.

## Materials and Methods

### Deposition of thin films

The co-deposition process including RF sputtering and RF-PECVD with supply power of 13.56 MHz was applied to fabricate Cu/Ni NPs thin films. This method is based on reactor with two electrodes different in material and size. The smaller one is metal as powered electrode while the bigger one is grounded by stainless steel chamber with 5 cm distance from each other. SiO_2_ substrates and Cu target were set into the chamber then at room temperature the chamber was evacuated to 10^3^ N/m^2^ as base pressure and acetylene gas was introduced to chamber then the pressure raised to the ambient. There are two main reasons for applying acetylene gas in this step, first one is its usage as carrier gas to plasma production and the second one is making NPs in the trace of carbon. Deposition process carried out at 3.5 N/m^2^ and 80 W as initial gas pressure and RF power respectively during 30 min. Then the vacuum was broken, and target was changed to Ni. Deposition process caried out again at 2.5 N/m^2^ and 150 W as initial gas pressure and RF power respectively. Finally, Cu and Ni NPs deposited under acetylene gas make Cu/Ni nanostructures. Details and ID of prepared samples are given in Table 1.

**Table 1:** Details and ID of prepared samples.

ID	Yield	Sputtering parameters			Sputtering time (min)
		basic pressure (n/m^2^)	Work pressure (N/m^2^)	Power (Watt)	
#1	Cu	10^3^	3.5	80	30
#2	Cu/Ni	10^3^	2.5	150	30/15
#3	Cu/Ni	10^3^	2.5	150	30/20

### Characterization of the films

A Nanoscope Multimode atomic force microscope (Digital Instruments, Santa Barbara, CA) in noncontact mode was applied to record 3D images of prepared samples in 1 μm × 1 μm scanning square areas while the scan speed was 10–20 μm/s. The MountainsMap® Premium software was used to process the 3D topographical maps obtained by AFM. Based on the ISO 25178-2:2012 standard^29–31^, several morphological parameters, explicitly, height, core, volume, feature, functional, spatial, and hybrid were recorded and discussed.

Using a Rutherford backscattering spectrometry (RBS) with high energy in order of MeV thickness and composition of prepared sample estimated. In the case of gas sensing operation, LSPR spectra was applied using a UV–Vis spectrometer in the range of 350 to 850 nm as wavelength while a typical sample is in the closed stainless-steel cell with diameter of 5.2 cm and height of 13.8 cm, under various flow of CO gas with purity of 99.9% based on IRSQ standard from Arian Gas Co. from 1.6 to 16 L/h during 180 s and 600 s. This step carried out at room temperature, ambient humidity of 19% and in a fume hood.

## Results and discussion

### Rutherford backscattering spectrometry

**Figure 1:**
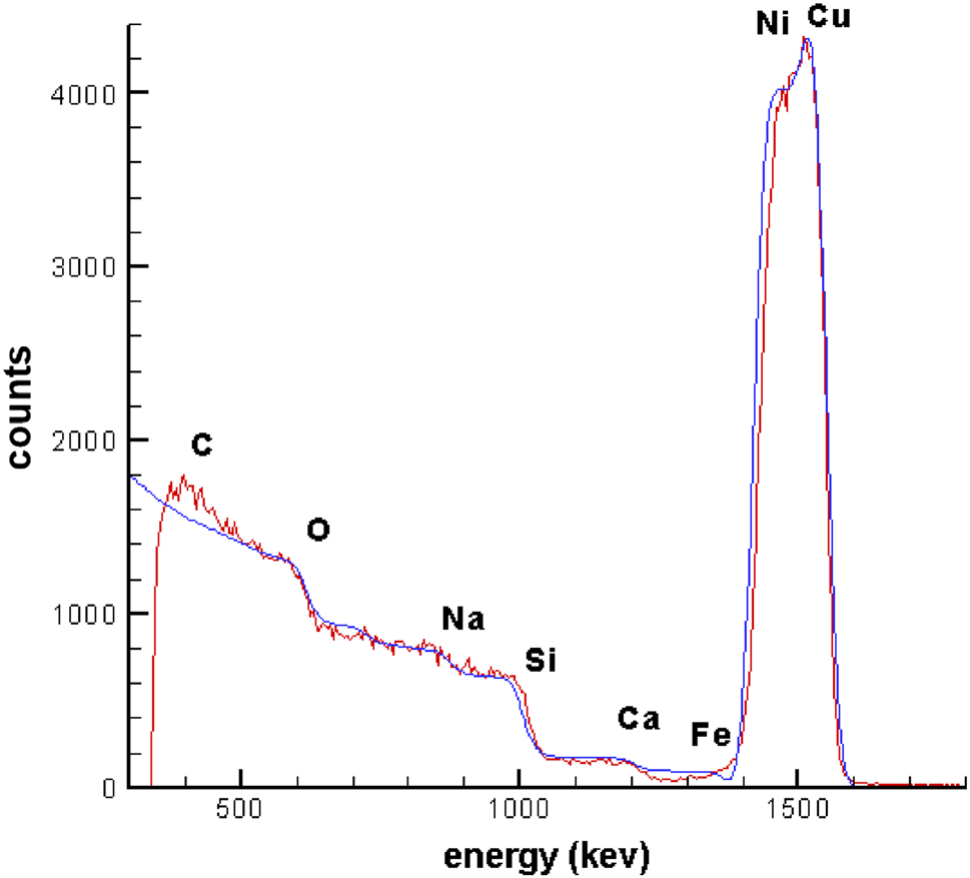
Experimental (red line) and simulated (blue line) RBS spectra of Cu/Ni sample.

Rutherford backscattering spectrometry as an ion scattering technique will be used for compositional thin film analysis. This unique method allows quantification without applying reference standards. In RBS analysis, directed high energy in order of MeV, (He^2+^ ions, i.e., α particles) onto sample and backscattered He^2+^ ions at a given angle will be measured. The SIMNRA code helps to simulate lines and curves whereas its agreement with experimental RBS spectra shows the quality of prepared sample. The RBS spectra of Cu/Ni NPs sample is shown in Fig. 1 which in the red line is experimental RBS spectra and the blue one is simulated by SIMNRA code, it can be seen that both spectra are in good agreement with each other. To identify the elements in our sample incident beam with 1985 keV was applied. The thickness of upper layer is about 40 1E15Atom/cm^2^ including 86% Ni, 0.10% O_2_, 0.02% C and 0.02% Fe. The Fe is related to impurity in Ni target during sputtering process. The peaks of Cu and Ni of bottom layer can be seen in 1500 keV and peaks of C and O_2_ in 426 keV and 582 keV respectively. The steps of Na, Si and Fe are in 870 keV, 983 keV, 1340 keV and 1823 keV respectively.

### 3D image analysis

#### Morphological aspects

**Figure 2:**
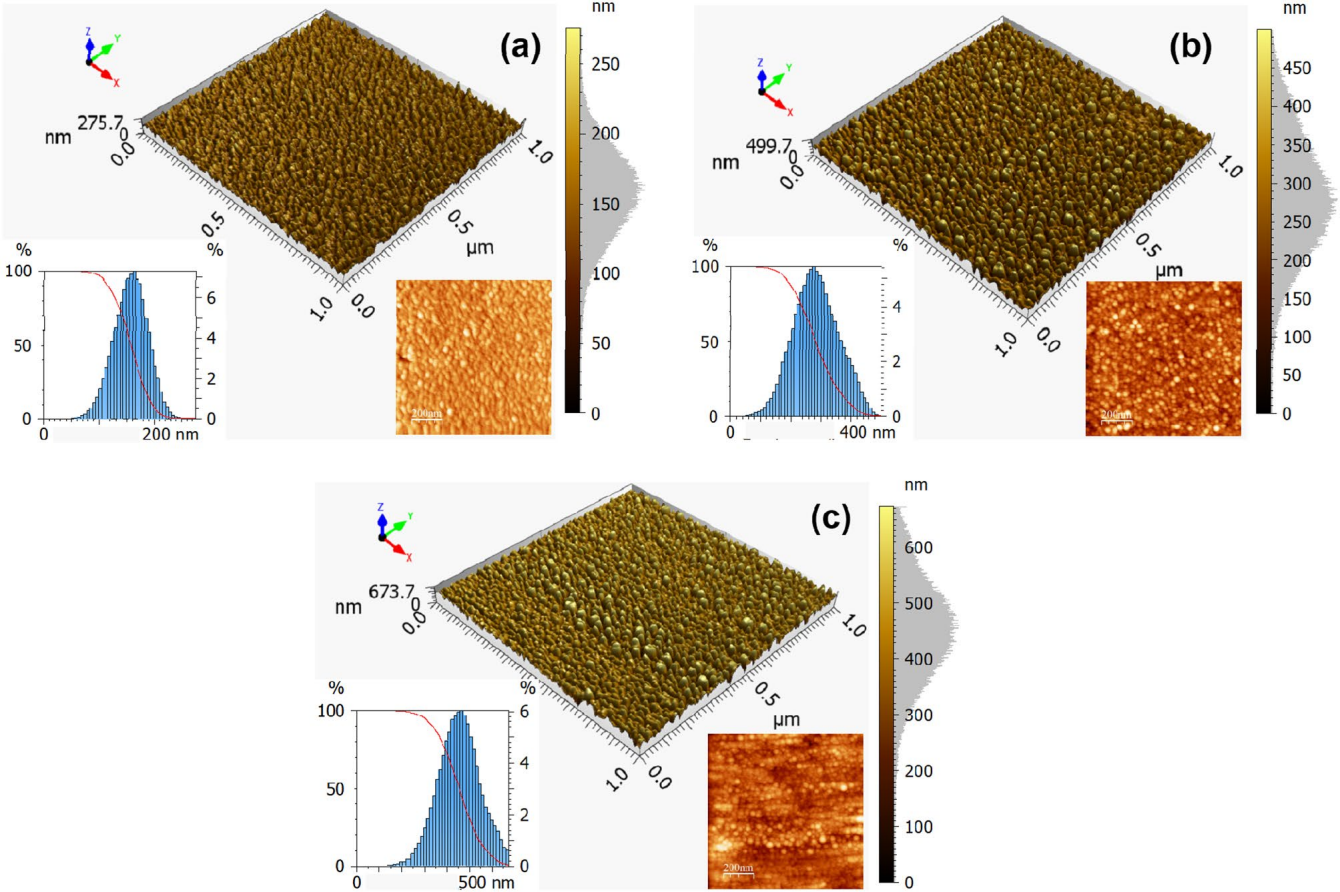
AFM maps of (**a**) Cu, (**b**) CuNi15, and (**c**) CuNi20 thin films. Their respective 2D maps, height distribution, and Abbott firestone curves are inset in each image.

The square AFM 3D topographic maps of the surface of Cu and Cu/Ni NPs thin films are shown in Fig. 2. Additionally, 2D topographic maps inserted into each figure reveal that the NPs observed on the film surface coalesced into a spherical shape, whereas such morphology present is similar to those reported by Ghodselahi and Arman^32^ and Arman et al.^33^. However, our Cu NPs did not agglomerate, and the sample containing only Cu displays an apparently smoother surface, whose rougher peaks are finer (Fig. 2a). Conversely, the peaks exposed on CuNi15 and CuNi20 samples have a pronounced spherical shape with higher intensity, as shown by the height scale in Fig. 2a and b. The visible changes in the morphology of the films suggest that the surfaces have different topographic spatial patterns, which were affected by the Ni deposition time.

**Figure 3:**
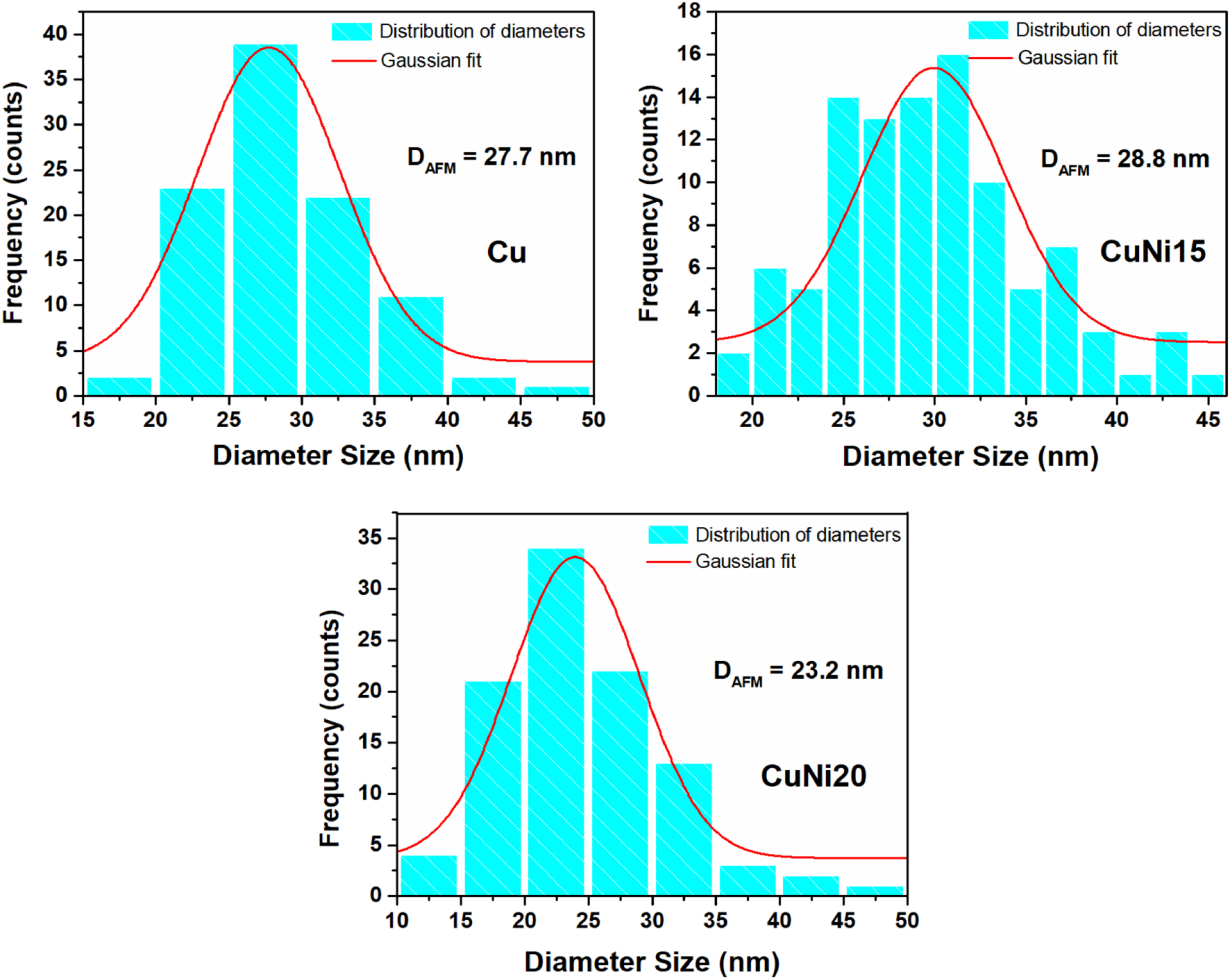
Particle diameter size distribution of (**a**) Cu, (**b**) CuNi15, and (**c**) CuNi20 thin films were obtained from the AFM topographical maps.

The average grain size of NPs was estimated using a Gaussian fit based on histograms of diameter distribution obtained from measurements of 100 NPs, as shown in Fig. 3. As can be seen, Cu and CuNi15 exhibited similar average grain sizes (27.7 and 28.8 nm), while CuNi20 has smaller grains (23.2 nm), which are values close to that reported by Ghodselahi et al.^34^ (around 24 nm). In bimetallic systems, a shift of the localized surface plasmon resonance peak can occur when there are changes in grain size^35^. In this regard, it can be concluded that high Ni deposition times can influence the surface plasmonic nature of Cu/Ni thin films for our systems.

The overall morphology also plays an important role in the spatial configuration of topographic patterns in thin films. Table 2 summarizes the height-based topographical parameters associated with AFM maps, which can be depicted by the temporal values of average roughness (Sa), Skewness (Ssk), and Kurtosis (Sku). The Sa values were found to be 1.12 (Cu), 3.17 (CuNi15), and 5.34 nm (CuNi20), confirming that the films become rougher as the Ni deposition time increases. These values are comparable to those previously reported by Arman et al.^33^ (1–4 nm), Ghodselahi et al.^34^ (1–1.05 nm), and Ţălu et al.^36^ (1.91–6.32 nm), where a similar sputtering method was used to the deposition of Cu/Ni NPs thin films. However, Ghosh et al.^37^ electrodeposited their Cu/Ni multilayers and reported higher roughness values, explicitly ranging from 13.8 to 36 nm. Notably, the difference in the surface formation dynamics for different deposition methods can lead to the formation of surfaces with different spatial patterns. Despite this, as can be seen, the RF-PECVD method is effective for the fabrication of Cu/Ni NPs thin films with a roughness not greater than 6.32 nm.

**Table 2:** Relevant height-based topographical parameters of the films.

Parameter	Unit	Cu	CuNi15	CuNi20
Sa	(nm)	1.12	3.17	5.34
Ssk	(–)	0.41	0.87	0.67
Sku	(–)	3.61	4.10	4.01

Regarding height profiles, the higher-order statistical moments Ssk and Sku are related to asymmetry and normality of the height distribution, respectively. All Ssk values are positive (Ssk > 0), showing that the right tail is longer^38^, as can be confirmed in the height distribution plots inset Fig. 2. Moreover, all height profiles have leptokurtic^39^ predominance (Sku > 3), proving that the height distribution curve is less flat than a gaussian curve for a normal distribution. The red line included in the height distribution plots is Abbott-Firestone curves^40^, a suitable statistical approach for evaluating normal data distribution. This line is obtained from the cumulative sums along the height histogram, where the highest peak and deepest valley are associated to its minimum (0%) and maximum (100%) values. These Abbott–Firestone curves expose a smoth S-like shape from the y-axis and for all cases suggest that from the roughest and most intense peak, the percentage of material traversed compared to the area spanned gradually increases. This confirms that the surfaces are dominated by spatial patterns that are affected by the Ni deposition time.

#### Advanced morphological analysis of CuNi 3D nanostructures

Table 3 summarizes the specific ISO morphological parameters associated with each surface obtained from AFM images. It is well-known that the area material ratio (Smr) and inverse area material ratio (Smc) are surface functional parameters^29^. For instance, our results showed that the area above the mid-plane of the surface is entirely covered by peaks in all films (Smr = 100%). However, the Smr values were obtained from different heights of the topographical bearing area ratio^41^, as the Smc parameter is known. The behavior of Smc is ascribed to the increase in the roughness of Cu → CuNi20, where it can be seen that the highest roughness value obtained for CuNi20 generates an Smc ~ 13 nm, while Cu exhibits a value of ~ 8 nm.

**Table 3:** ISO surface parameters were obtained from the AFM topographical maps of the films.

Parameter	Unit	Cu	CuNi15	CuNi20
Smr	(%)	100	100	100
Smc	(nm)	8.43	10.5	12.7
Sdq	(–)	7.11	17.02	21.30
Sdr	(%)	229	778	945
Spd	(1/µm^2^)	436	557	755
Spc	(1/µm)	612	782	925
Sk	(nm)	8.47	19.50	23.7
Spk	(nm)	5.01	16.17	22.4
Svk	(nm)	7.80	13.80	23.64
Vmp	(µm^3^/µm^2^)	1.29 × 10^–3^	2.99 × 10^–3^	3.86 × 10^–3^
Vmc	(µm^3^/µm^2^)	2.81 × 10^–3^	68.59 × 10^–3^	82.65 × 10^–3^
Vvc	(µm^3^/µm^2^)	36.10 × 10^–3^	97.69 × 10^–3^	114.0 × 10^–3^
Vvv	(µm^3^/µm^2^)	3.67 × 10^–3^	7.80 × 10^–3^	10.64 × 10^–3^

The hybrids parameters root mean square gradient (Sdq) and developed interfacial area ratio (Sdr) are parameters associated with the flatness and texture complexity. The Sdq values range 7–21 from Cu → CuNi20, showing an increase in the topographic irregularity in the films when the Ni layer is deposited within 20 min of duration. Notably, the surface of CuNi20 is less flat than the surface of Cu. Furthermore, the values of Sdr, a surface microtexture complexity-related parameter, were found to increase from Cu → CuNi20. According to Kamble et al.^42^, the surface microtexture complexity increases when Sdr increases, indicating herein that CuNi20 (Sdr = 945%) has a more complex surface microtexture compared to Cu film (Sdr = 229%). Indeed, the change in the complexity of the microtexture played a key role in the distribution and shape of the rough peaks, as can be observed for the feature parameters density of peaks (Spd) and arithmetic mean peak curvature (Spc). In this regard, Spd increased from Cu → CuNi20, showing that the peaks organize more closely together as the Ni layer thickness increases. In addition, Spc also increased from Cu → CuNi20, revealing that the shape of the peaks on the surface of Cu samples are more rounded (Spc = 612), while for CuNi20 they are more pointed (Spc = 925).

The rough profile of each film also exhibits different spatial patterns in the peak, core, and valley regions of the surface. The core (Sk), reduced peak (Spk) (above core), and valley (Svk) (below core) heights^31,43^, which are parameters measured perpendicularly to the surface plane^30^, increase from Cu → CuNi20, as a result of the notable increase of the surface roughness. Similarly, the peak material (Vmp), core material (Vmc), dale void (Vvv), and core void (Vvc) volumes^31^ present the same trend, as all values increased from Cu → CuNi20. Such behavior indicates that the CuNi20 surface can retain more fluid than the other samples, which is a positive factor that suggests that this surface can be lubricated more easily^44^. Thus, it is notable that the modification of the topographic profile as the Ni layer thickness increases from CuNi15 → CuNi20 is behind these changes in the advanced morphological parameters, affecting the surface microtexture and spatial patterns of the films.

#### 3D qualitative surface texture analysis

**Figure 4:**
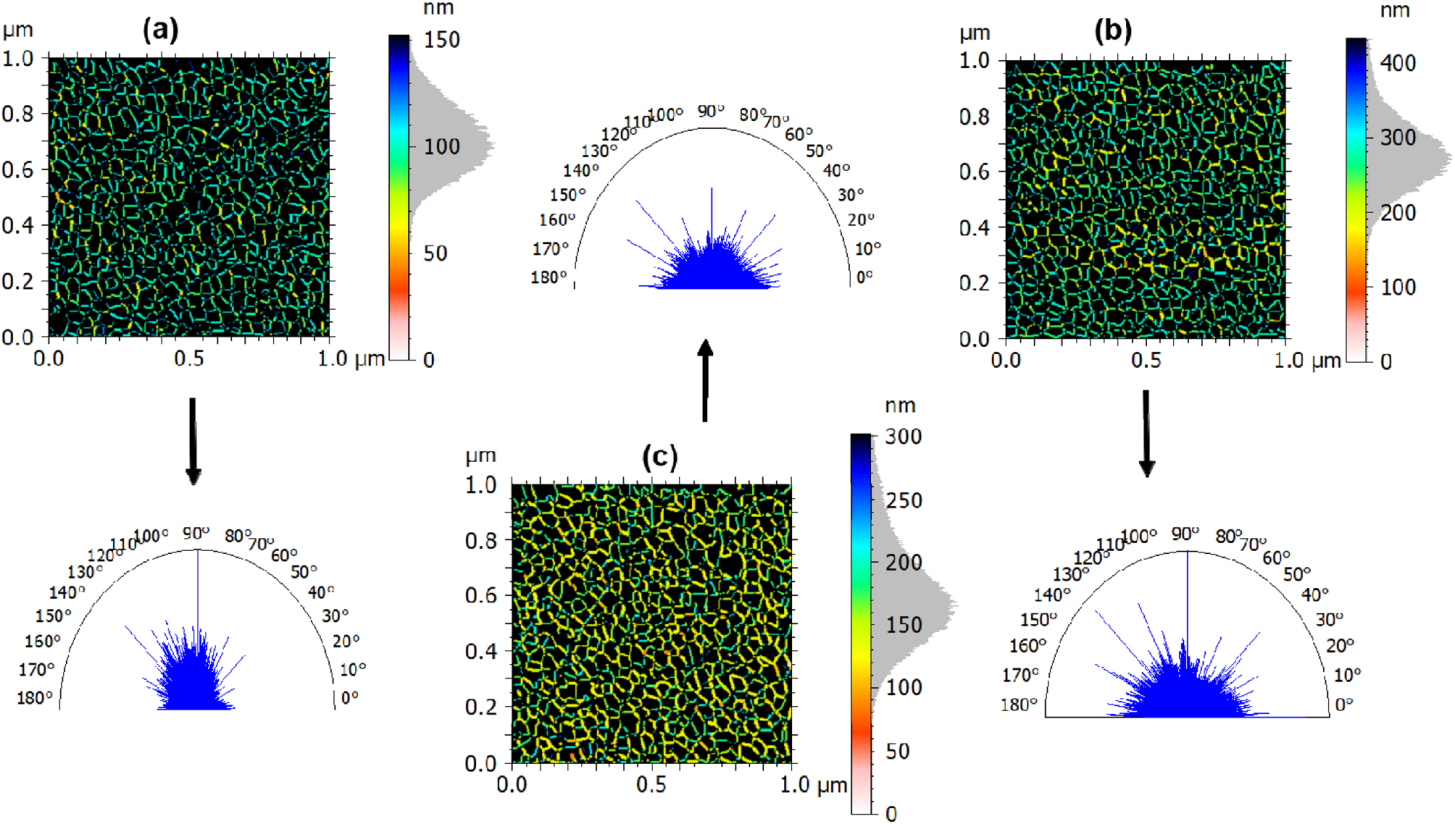
Furrows and surface isotropy of the surface microtexture of (**a**) Cu, (**b**) CuNi15, and (**c**) CuNi20 thin films.

A qualitative assessment of the surface microtexture of the films was obtained by renderings from the AFM topographical maps using MountainsMap^45^ commercial software. The renderings are exposed in Fig. 4 and show representative furrows and polar graphs associated with the surfaces. The furrows and spatial parameters are summarized in Table 4. The furrow images reveal that the samples are dominated by similar channel systems with apparent furrow uniformity. However, both maximum depth of furrows (MDF) and Mean depth of furrows (MDEF) parameters increased from Cu → CuNi20, confirming previous observations on the lubricating potential of CuNi20. It is worth noting that Cu (Fig. 4a) and CuNi15 (Fig. 4b) samples show substantially similar color scales, suggesting that the surface microtexture of the Cu film does not change significantly with a layer of Ni film deposited for 15 min. In contrast, the sample CuNi20 (Fig. 4c) exhibits furrows with robust different color scales, ascribed to its higher values of MDF and MDEF.

**Table 4:** Surface microtexture parameters of the films.

Parameter	Unit	Cu	CuNi15	CuNi20
MDF	(µm)	0.08	0.07	0.11
MDEF	(µm)	0.05	0.06	0.09
Ti	(%)	47.87	80.23	81.13
Sal	(nm)	1.34	2.29	4.75
Std	(°)	121	2.50	3.52

The polar graphs included in Fig. 4 also show that the surface microtexture is different. Notably, the deposition of the Ni layer significantly changes the spatial patterns. The microtexture isotropy of the samples was computed to be 48 (Cu), 80 (CuNi15), and 81% (CuNi20). As can be noticed, the Ni layer deposition promotes the formation of more isotropic microtextures, while the single Cu film has a more anisotropic surface microtexture. Moreover, CuNi15 and CuNi20 are dominated by low dominant spatial frequencies because they present large values of the autocorrelation length (Sal)^44^ compared to Cu sample. This is also combined with the similar texture directions exhibited by these samples (Std = 2.5° and Std = 3.5°), while Cu sample recorded a robust large value (Std = 121°). Based on these results, all films exhibited long-range spatial changes due to the different morphologies, topographical profiles, and roughness presented. Therefore, these results prove that the Ni layer deposition time plays an important role in forming bimetallic sputtered CuNi surfaces.

### Spectroscopic analysis

**Figure 5:**
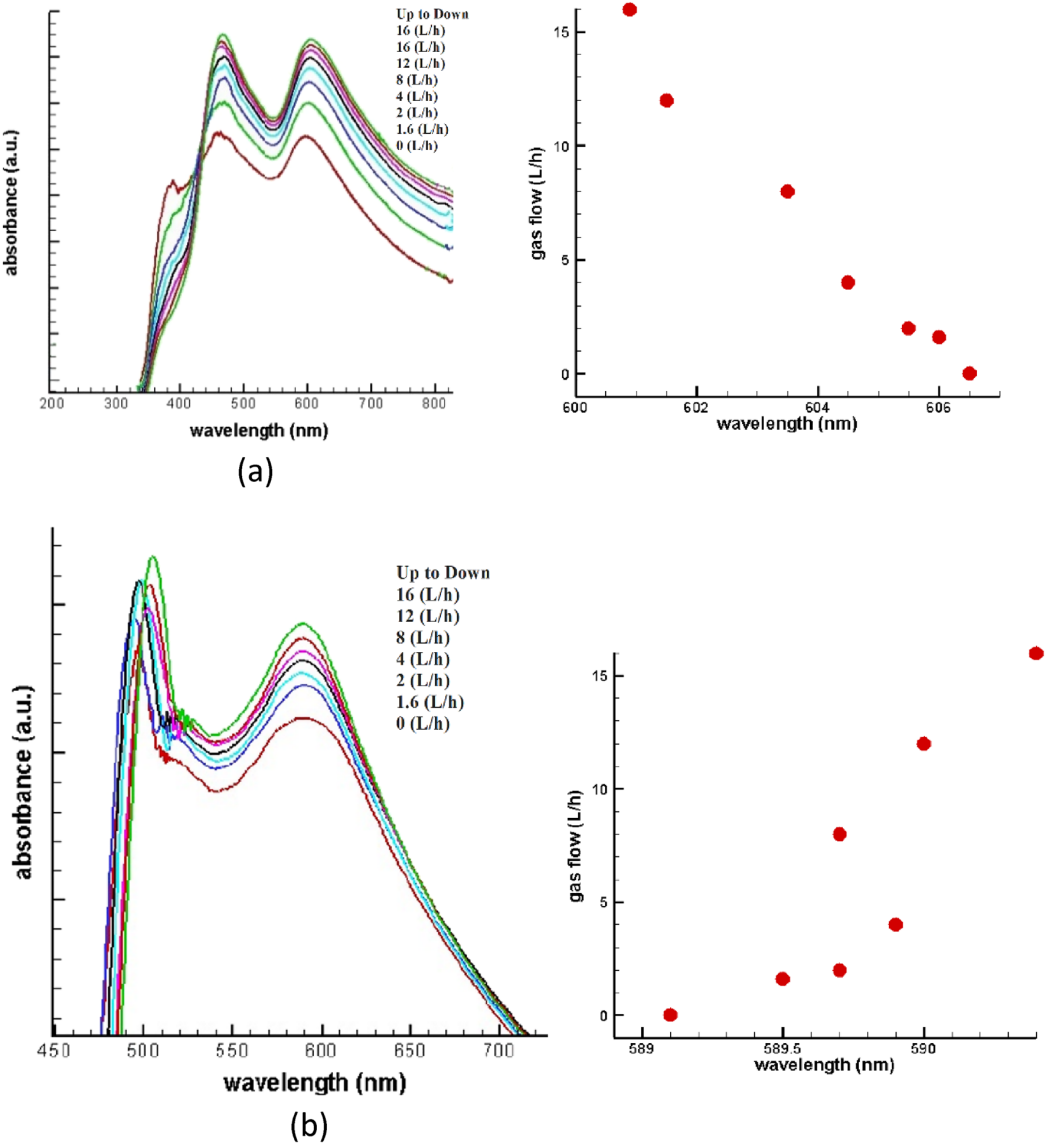
Absorption spectra of NPs under air and CO flow, position of plasmonic peak versus gas flow for a) CuNi15 and b) CuNi20.

To study the LSPR behavior of Cu/Ni NPs under air and different CO gas fluxes at room temperature, UV–visible absorption spectra in the wavelength range of 350–800 nm was applied which is shown in Fig. 5 for CuNi15 and CuNi20. By introducing different fluxes of CO gas, the effective LSPR peak of CuNi15 is going to be wider with more intensity in absorbance besides, the peaks shift to higher wavelengths (red shift) from 597.5 nm under air flow to 606.0 nm under 16 L/h CO flow for 180 s and 606.5 nm under 16 L/h CO flow for 600 s. On the other hand, CuNi20 shows different behavior so that increasing the flow of CO gas leads to decreasing the position of LSPR peaks in wavelength (blue shift) from 600.0 nm under air flow to 589.5 nm under 16 L/h CO flow for 180 s and 589.1 nm under 16 L/h CO flow for 600 s. The same as CuNi15 we can see the wider peaks and increasing intensity in absorbance for CuNi20. It can be estimated that by increasing the thickness of Ni layer on Cu and increasing the size and number of NPs in CuNi20 rather than CuNi15, Cu and Ni particles get closer then, the amplitude of electrons oscillation increases so on the frequency increases it means that the wavelength decreases, and blue shift happens.

**Table 5:** Position of plasmonic peak according to gas flow and time of sensing.

Gas flow (L/h)	0	1.6	2	4	8	12	16	16
Time of introducing gas (s)	180	180	180	180	180	180	180	600
CuNi15 Position of plasmonic peak (nm)	597.5	600.9	601.5	603.5	604.5	605.5	606.0	606.5
CuNi20 Position of plasmonic peak (nm)	600.0	590.4	590.0	589.7	589.9	589.7	589.5	589.1

Table 5 shows the wavelength of plasmonic peaks based on gas flow and time of sensing for CuNi15 and CuNi20 in detail. Increasing time from 180 to 600 s with constant gas flow of 16 L/h causes a small change in position of plasmonic peak, which can be associated to the decrease in grain size observed in the morphological analysis. The decrease in the grain size of CuNi15 → CuNi20 promoted a change in the general topographical. As a consequence, the topographic profile became rougher and more isotropic. According to Yang et al.^46^, the morphology geometry and surface texture isotropy can affect the surface plasmon. In this regard, the shift of the wavelength of plasmonic peaks occurred due to a change in morphology and an increase in topographic roughness and surface texture isotropy. Therefore, the localized surface plasmon can be controlled by the adjust of morphological aspects in bimetallic CuNi systems.

## Conclusion

In summary, Cu/Ni NPs at the trace of acetylene as carbon source was deposited separately using RF-PECVD technique from Cu and Ni targets. Experimental and simulated spectra of RBS confirm each other, indicating the quality of prepared sample. AFM images showed different topographic profiles generated by different Ni deposition times. The decrease in grain size for the longer Ni deposition time is behind the higher surface roughness found. Despite this, a flatter surface was observed by the sample formed after 20 s of deposition. This sample was also assigned to be the most isotropic, as a result of the smaller grain size and rougher topographical profile. The red shift of localized surface plasmonic resonance was studied by absorption spectra and indicated the gas sensing effect of Cu/Ni NPs. Such effect was tuned by decreasing grain size and increasing topographical texture isotropy compared to a single Cu film. Our findings prove that the localized surface plasmon of layer-by-layer Cu/Ni thin films can be optimized by their grain size and morphological aspects, which can also be of great interest para (CO) gas chemical sensing applications.

## Data Availability

The data that support the findings of this study are available from the corresponding author upon reasonable request.
